# A102 INTERDISCIPLINARY TELEHEALTH REFERRAL PATHWAY AND CONSULTATIONS TO IMPROVE OUTCOMES AMONG CANADIAN OLDER ADULTS WITH LIVER CIRRHOSIS

**DOI:** 10.1093/jcag/gwac036.102

**Published:** 2023-03-07

**Authors:** J Zhu, F Carr, P Tian, M McLeod, M MacFarlane, S De Coutere, M Sun, K Peltekian

**Affiliations:** 1 Digestive Care and Endoscopy, Dalhousie University, Halifax; 2 Geriatric Medicine; 3 Family Medicine, University of Alberta, Edmonton; 4 General Internal Medicine; 5 Nursing; 6 Geriatric Medicine, Dalhousie University, Halifax, Canada

## Abstract

**Background:**

Telehealth and telemedicine have become indispensable healthcare delivery tools during the COVID-19 pandemic. Older individuals with cirrhosis have complex medical needs that are currently unmet due to the growing disease burden and decreased access to care. Delivering timely specialist care virtually to older adults with cirrhosis will likely be beneficial and acceptable to such patients; however, this has not yet been prospectively evaluated.

**Purpose:**

The primary goal is to pilot the delivery of dual specialist care from a hepatologist and geriatrician, delivered virtually, for older adults living with liver cirrhosis who are at high risk of geriatric syndromes (age >/= 65 with frailty, undifferentiated cognitive impairment from dementia or hepatic encephalopathy, recurrent falls, risk factors for polypharmacy and moderate to severe malnutrition). Care is delivered using a dedicated hepatology-geriatric referral pathway. Primary objectives include evaluating the impact of this approach on emergency care and inpatient utilization, along with patient attitude and satisfaction to the virtual interdisciplinary care delivery model.

**Method:**

This pilot quality improvement study was conducted in Halifax, Nova Scotia. Ethics approval was obtained from the Nova Scotia Health Research Ethics Board and the University of Alberta Research Ethics Board. Fifty to one hundred participants (age 65 years or older with at least one geriatric syndrome; diagnosis of liver cirrhosis by liver elastography or liver biopsy, or Fibrosis-4 Index for Liver Fibrosis greater than three and having radiological features of cirrhosis and/or portal hypertension) were recruited between September 2022 to December 2022 at the time of their hepatology consultation. After consent and screening, each patient underwent a telehealth appointment by zoom with a geriatrician within four weeks of their initial hepatology assessment. Follow-up by telephone using a standardized survey regarding ease of access and quality of their telehealth experience then occurred at 3-4 weeks, 3 months and 6 months for emergency room visits and hospital admission status.

**Result(s):**

Pending

**Conclusion(s):**

Pending

**Please acknowledge all funding agencies by checking the applicable boxes below:**

Other

**Please indicate your source of funding;:**

Pfizer Canada

**Disclosure of Interest:**

J. Zhu Grant / Research support from: Pfizer Canada, F. Carr Grant / Research support from: Pfizer Canada, P. Tian: None Declared, M. McLeod: None Declared, M. MacFarlane: None Declared, S. De Coutere: None Declared, M. Sun: None Declared, K. Peltekian: None Declared

